# RESOLVE-DWI-based deep learning nomogram for prediction of normal-sized lymph node metastasis in cervical cancer: a preliminary study

**DOI:** 10.1186/s12880-022-00948-6

**Published:** 2022-12-17

**Authors:** Weiliang Qian, Zhisen Li, Weidao Chen, Hongkun Yin, Jibin Zhang, Jianming Xu, Chunhong Hu

**Affiliations:** 1 Department of Radiology, The First Affiliated Hospital of Soochow University, No.188 Shizi Street, Suzhou, 215006 Jiangsu People’s Republic of China; 2 Department of Radiology, The Affiliated Suzhou Hospital of Nanjing Medical University, No.26 Daoqian Street, Suzhou, 215002 Jiangsu People’s Republic of China; 3 Beijing Infervision Technology Co., Ltd, No.60 Dongsihuan Middle Road, Chaoyang District, Beijing, 100020 People’s Republic of China

**Keywords:** Cervical cancer, Lymph node metastasis, Deep learning, Diffusion weighted imaging

## Abstract

**Background:**

It is difficult to predict normal-sized lymph node metastasis (LNM) in cervical cancer clinically. We aimed to investigate the feasibility of using deep learning (DL) nomogram based on readout segmentation of long variable echo-trains diffusion weighted imaging (RESOLVE-DWI) and related patient information to preoperatively predict normal-sized LNM in patients with cervical cancer.

**Methods:**

A dataset of MR images [RESOLVE-DWI and apparent diffusion coefficient (ADC)] and patient information (age, tumor size, International Federation of Gynecology and Obstetrics stage, ADC value and squamous cell carcinoma antigen level) of 169 patients with cervical cancer between November 2013 and January 2022 were retrospectively collected. The LNM status was determined by final histopathology. The collected studies were randomly divided into a development cohort (n = 126) and a test cohort (n = 43). A single-channel convolutional neural network (CNN) and a multi-channel CNN based on ResNeSt architectures were proposed for predicting normal-sized LNM from single or multi modalities of MR images, respectively. A DL nomogram was constructed by incorporating the clinical information and the multi-channel CNN. These models’ performance was analyzed by the receiver operating characteristic analysis in the test cohort.

**Results:**

Compared to the single-channel CNN model using RESOLVE-DWI and ADC respectively, the multi-channel CNN model that integrating both two MR modalities showed improved performance in development cohort [AUC 0.848; 95% confidence interval (CI) 0.774–0.906] and test cohort (AUC 0.767; 95% CI 0.613–0.882). The DL nomogram showed the best performance in development cohort (AUC 0.890; 95% CI 0.821–0.938) and test cohort (AUC 0.844; 95% CI 0.701–0.936).

**Conclusion:**

The DL nomogram incorporating RESOLVE-DWI and clinical information has the potential to preoperatively predict normal-sized LNM of cervical cancer.

## Background

Cervical cancer is one of the most common gynaecological malignancies worldwide [1]. Lymph node metastasis (LNM) is closely associated with the prognosis of patients with cervical cancer and may lead to poorer 5 year overall survival and 5 year recurrence-free survival [2]. In general, pathology is the gold standard for determining LNM status. However, invasive examinations are not routine clinical methods due to the associated complications such as symptomatic postoperative lymphocele and lower extremity lymphedema [3]. The latest International Federation of Gynecology and Obstetrics (FIGO) guidelines have revised the staging system for cervical cancer [4]. It is worth noting that radiological evaluations of LNM have been incorporated into the staging system for the first time. Thus, accurate preoperative predictions of LNM status are helpful for clinical decision-making.

Preoperative imaging evaluation primarily identifies LNM using size criteria (≥ 10 mm in the short axis), which often leads to low sensitivity due to the inability to discriminate normal-sized metastatic lymph nodes [5, 6]. Unfortunately, more than half of metastatic lymph nodes are less than 10 mm in clinical practice [7]. This means that routine imaging methods have extreme difficulties in identifying such normal-sized LNM.

With the rapid development of quantitative image analytics, researchers have changed from relying only on image signs to focusing on semantic features extracted from image data [8]. Among these efforts, deep learning (DL) is one of the most promising technologies in the field of medical imaging. DL can transform image analysis via automated discovery of salient feature representations for different tasks [9]. DL has been applied to predict LNM using different medical images of different tumours, including in predicting normal-sized LNM [10–13].

Magnetic resonance imaging (MRI), and diffusion-weighted imaging (DWI) in particular, which can reflect the motion of water molecules constrained by tissue ultrastructures with a quantitative parameter of apparent diffusion coefficient (ADC) value and enable noninvasive detection of small LNMs [14], is recommended for the initial evaluation of cervical cancer. However, we noted that single-shot echo-planar imaging (SS-EPI) has mostly been used in studies extracting DWI information to predict LNM in cervical cancer [15, 16], when it has been reported that conventional DWI based on SS-EPI has poor image quality because of its susceptibility to artefacts and geometric distortion; in contrast, RESOLVE-DWI improves image quality and offers accurate ADC values [17, 18]. Therefore, the aim of our study was to investigate the feasibility of using RESOLVE-DWI-based DL nomogram to preoperatively predict normal-sized LNM in cervical cancer.

## Methods

### Study population

This study was approved by the institutional ethics committee of our hospital. Between November 2013 and January 2022, a total of 274 cervical cancer patients who underwent MRI examination (including RESOLVE-DWI) within 2 weeks before surgery was retrospectively retrieved and the informed consent requirement was waived. The exclusion criteria were as follows: (1) preoperative adjuvant chemotherapy or radiotherapy (n = 15); (2) did not undergo lymphadenectomy and the LNM status was not available (n = 23); (3) enlarged (≥ 10 mm in the short axis) metastatic lymph nodes (n = 27); (4) cancers with FIGO stage earlier than IB (n = 21); (5) unavailable or incomplete clinicopathologic information (n = 17); (6) insufficient image quality (n = 2). Finally, there were 169 patients (41 metastasis and 128 non-metastasis) enrolled in this study.

### MRI acquisition

All MR examinations were performed on a 3T MR scanner (MAGNETOM Skyra, Siemens Healthcare, Erlangen, Germany) with an 18-channel surface coil in combination with a 32-channel spine coil. The axial RESOLVE-DWI parameters: repetition time/echo time (TR/TE): 5300/117 ms; field of view (FOV): 240 × 240 mm^2^; slice thickness: 7 mm; slice gap: 0 mm; average: 2; matrix: 130 × 130, b values (0 and 800 s/mm^2^). ADC maps were automatically calculated on a commercial workstation (Syngo via, Siemens Healthcare, Erlangen, Germany) from the RESOLVE-DWI.

### Data pretreatment

The resolution of the RESOLVE-DWI and ADC sequence MR images were reset to 512 × 512 prior to model development. No alignment was applied to the MR images, thus the 3D tumor regions of MR images were manually labeled separately by a radiologist with 8 years of experience using the open-source ITK-SNAP software (version 3.8.0, http://www.itksnap.org). The segmentations were further confirmed by another radiologist with 20 years of experience.

Clinical information was extracted from radiological records and biochemistry test results, and was recorded in relation to model development as followings: (1) age, which was an actual variable; (2) tumor size, defined as the greatest tumor diameter measured on MRI, which was a trichotomous categorical variable (< 2 cm = 1, 2– 4 cm = 2, > 4 cm = 3); (3) FIGO stage, which was a trichotomous categorical variable (IB = 1, IIA = 2, IIB = 3); (4) ADC value, recorded as the mean ADC value at the largest level of the tumor on ADC map, which was an actual variable; (5) squamous cell carcinoma antigen (SCCa) level, which was an actual variable.

### Development of the DL models

**Fig. 1:**
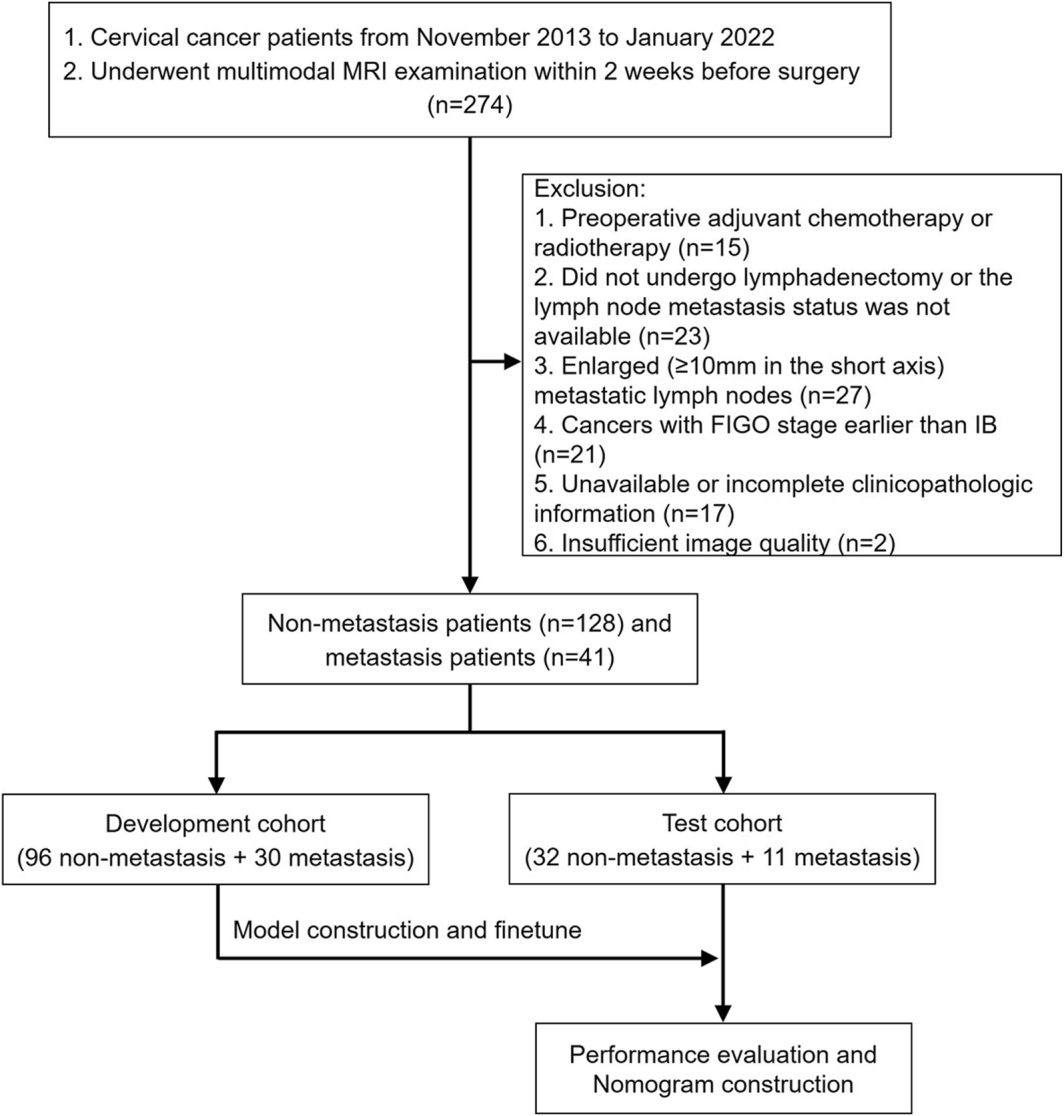
Study workflow in the development and evaluation of DL models for normal-sized LNM prediction of cervical cancer

During the implementation of the DL models, the patients were randomly divided into a development cohort (96 non-metastasis and 30 metastasis) and a test cohort (32 non-metastasis and 11 metastasis) at the ratio of 3:1. The DL models were trained and finetuned in the development cohort, and the test cohort was the held-out dataset and was never used before performance evaluation. The study workflow was depicted in Fig. 1.

Based on the size of the largest tumor region of all patients, the images from each case contained two modalities of MRI (RESOLVE-DWI and ADC maps) were transformed for the development of the DL models and defined as followings: (1) A 3D cube of 128 pixel × 128 pixel × 12 layers containing tumor regions cropped from the original images, while the peri-tumoral areas were padding with zero. (2) A 3D cube of 128 pixel × 128 pixel × 12 layers containing only peri-tumoral regions, while the tumor ROI was padding with zero. (3) The pathologically identified metastasis status label of each patient.

Given that the data size was relatively small for DL, data augmentation was performed to improve the model performance and reduce the risk of overfitting. The data augmentation approaches used in this study included scaling (0.8, 0.9, 1.1 or 1.2 times the image size), translation (up and down, left and right, front and back) and mirroring. The sample size in the development cohort increased to 9 times that of the original data.

A deep convolutional neural network (CNN) architecture named ResNeSt was used as the backbone of the DL models [19]. Particularly, the utilization of ResNeSt in this study aimed to exploit its novel Split-Attention block to improve the learned feature representations of the status of LNM in cervical cancer, which could be hardly identified by radiologists with the visual characteristics.

**Fig. 2:**
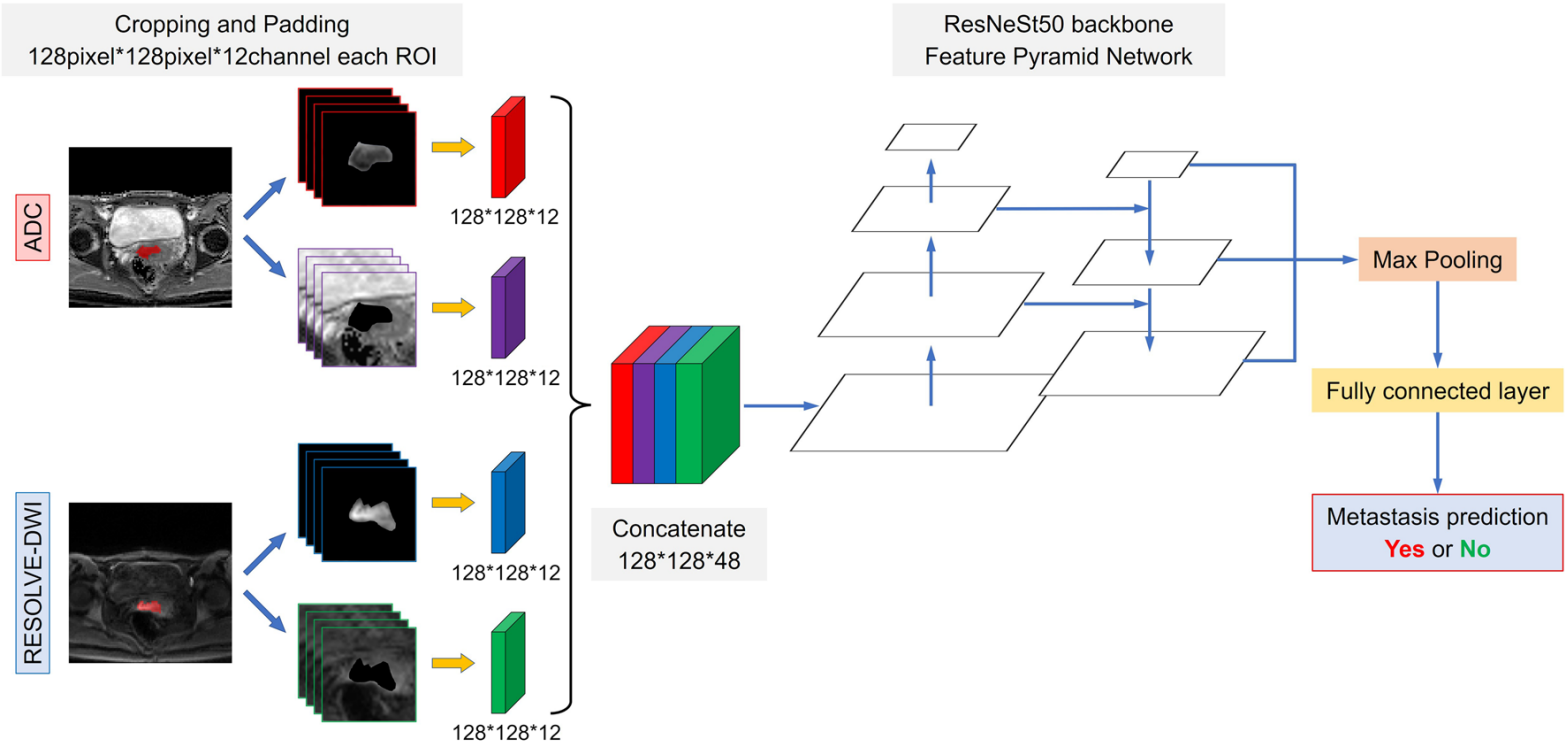
Schematic illustration of the combined model

Two kinds of DL models were proposed for our purpose. A single-channel CNN using the RESOLVE-DWI modality of the MR images (DWI model) or the ADC modality of the MR images (ADC model) as input, and a multi-channel CNN that integrating both modalities of the MR images (Combined model). For the single-channel CNN model, the 3D cubes containing the tumor ROIs and the peri-tumoral regions of each patient were concatenated and finally a 128*128 matrix with 24 layers (12 layers from the tumor ROIs and 12 layers from peri-tumoral regions) were generated as input image. For the multi-channel CNN model, the input images of the RESOLVE-DWI modality and the ADC modality were further concatenated into a 128*128 matrix with 48 layers (24 layers from the RESOLVE-DWI modality and 24 layers from the ADC modality) and used as input image. The conceptual architecture of the combined model was shown in Fig. 2.

To improve the model robustness and generalization, transfer learning technique was used. The ResNeSt network used in the DL models were pre-trained on the ImageNet dataset and multiple medical image datasets including TCGA-LUAD and TCGA-LUSC datasets from The Cancer Imaging Archive (TCIA) database [20], as well as the BraTS dataset from the Kaggle competition (https://www.kaggle.com/awsaf49/brats20-dataset-training-validation). The stochastic gradient descent optimizer was implemented in the training stage with momentum of 0.9 and weight decay of 0.0001. The minibatch size was 16 and the dropout rate was 0.5, other parameters were set to their default values. A weighted oversampling technique was also applied, only the resampled minibatches with a non-metastasis/metastasis ratio between 1.5:1 and 1:1.5 was selected for training. The proposed networks were trained based on the cross-entropy loss function and area under the curve (AUC) was used as metric for evaluation. In addition, we used cosine annealing strategy as the learning rate schedule to train our models more effectively. The initial learning rate was set to 0.0125 and warmed up to the fifth epoch, with followed cosine annealing to 120 epochs. The training was stopped when the loss function was stable. During model construction, 5-fold cross-validation was applied to avoid overfitting, and we used weighted ensemble method to integrate a weighted average result from those cross-validation models.

The development and test of the DL models were performed with python version 3.5 on the InferScholar platform (version 3.5, InferVision, China) [21].

### Model interpretability

To better understand the most influential regions in the MR images of our DL model, we had used a visual explanation tool named Gradient-weighted Class Activation Mapping (Grad-CAM) to improve the interpretability of the combined model. The saliency maps were obtained by applying Grad-CAM on the last convolutional layer of the neural network. Inspired by the generation of class activation maps with a “fine-tune” procedure [22], the trained combined model was used to fine-tune the same network taking single modality data as input and the corresponding heatmaps of the Resolved-DWI or ADC sequences were generated by using the fine-tuned single-modality models.

### Nomogram construction

To develop a clinically applicable method that could predict the normal-sized LNM probability of a cervical cancer patient, a nomogram was constructed by incorporating the following candidate variables: age, tumour size, FIGO stage, ADC value, SCCa level and the DL score on the basis of the multivariate regression analysis in the development cohort. The diagnostic performance of the nomogram was evaluated by receiver operator characteristic curve (ROC) analysis in the test cohort. In addition, the decision curve analysis (DCA) was plotted to assess the clinical usefulness of both the nomogram and the DL models.

### Statistical analysis

The differences of clinical variables with a continuous distribution were assessed by independent sample t-test or Mann-Whitney U test, and Chi-square test or Fisher’s exact test were used to evaluate the differences for categorical variables. The performance of predictive models was evaluated by the ROC analysis and the AUC. Delong’s test was used to compare the AUCs between different models. The development of nomogram was performed using the “rms” package. The DCA curve was plotted using the “dca.R” package. All the statistical analyses were performed via SPSS 23.0 (IBM) and R statistical software (https://www.r-project.org), and a two-sided P value < 0.05 was considered statistically significant.

## Results

### Patient characteristics

The clinicopathologic characteristics of enrolled patients were summarized in Table 1. There were no significant differences in terms of LNM prevalence between the development cohort and the test cohort [23.8% (30/126) and 25.6% (11/43), *P* > 0.05]. The mean age and FIGO stage between patients with LNM and non-metastasis showed no significant differences in both cohorts (all *P* > 0.05). The tumor size of metastasis group was significantly larger than that of the non-metastasis group in both cohorts (both *P* < 0.05). The lower ADC value of primary tumor was found in the metastasis group in both cohorts (both *P* < 0.05). The serum SCCa level significantly increased in the metastasis group in both cohorts (both *P* < 0.05).

**Table 1:** Clinicopathological characteristics of the cervical cancer patients

	Development cohort			Test cohort		
	Non-metastasis (n = 96)	Metastasis (n = 30)	*P*-value	Non-metastasis (n = 32)	Metastasis (n = 11)	*P*-value
Age (years)	50.45 ± 10.59	52.57 ± 9.54	*0.330*	53.16 ± 9.73	50.64 ± 7.19	*0.436*
Tumor size			*0.008*			*0.035*
< 2 cm	23	2		9	0	
2–4 cm	44	10		17	5	
> 4 cm	29	18		6	6	
FIGO stage			*0.219*			*0.278*
IB	68	17		27	7	
IIA	24	10		4	3	
IIB	4	3		1	1	
ADC value (×10^− 3^mm^2^/s)	0.96 ± 0.14	0.89 ± 0.12	*0.023*	0.98 ± 0.14	0.86 ± 0.11	*0.015*
SCCa level (ng/mL)	4.51 ± 7.38	11.32 ± 13.80	*0.001*	3.93 ± 4.76	8.79 ± 6.46	*0.007*

*FIGO* International Federation of Gynecology and Obstetrics; *ADC* Apparent diffusion coefficient; *SCCa* Squamous cell carcinoma antigen

*P* < 0.05 indicates statistically significant

## Performance of the DL models

**Fig. 3:**
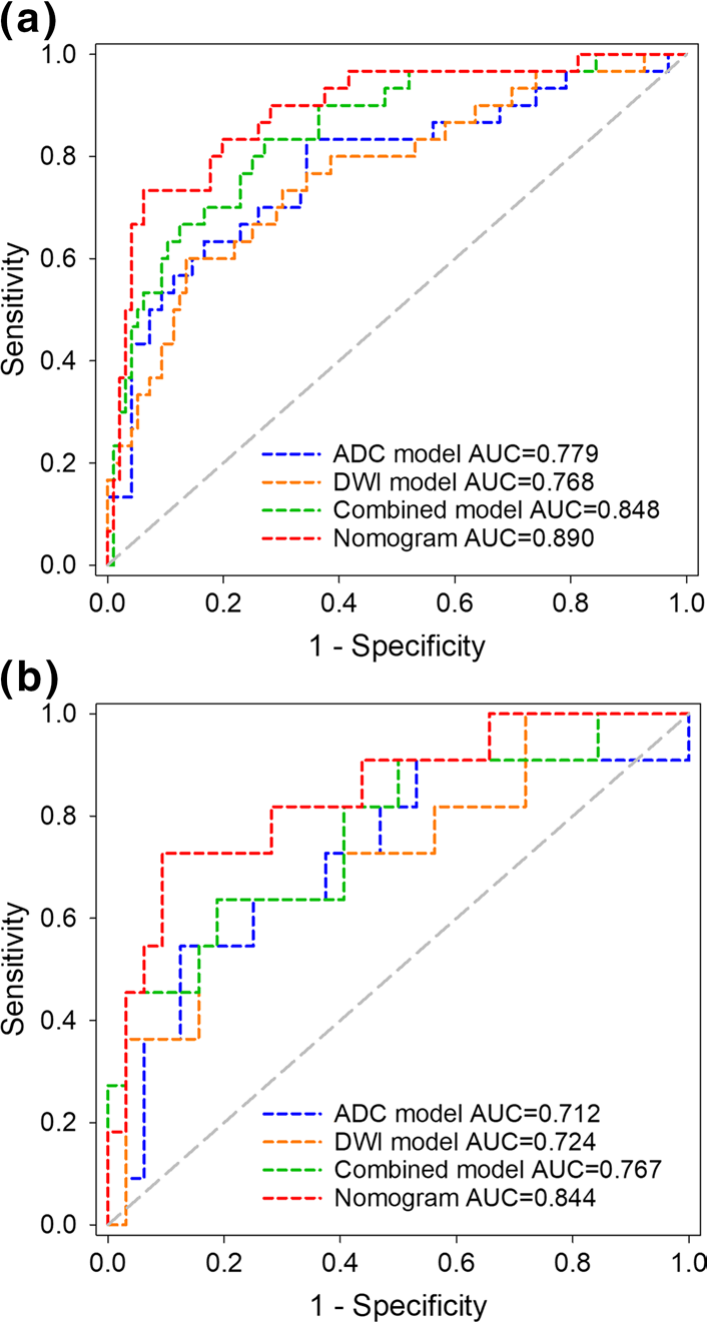
The ROC analysis comparison of the ADC model, DWI model, combined model and nomogram in the development cohort (**a**) and test cohort (**b**)

For the models using single modality MR images, the AUCs of the DWI model and ADC model were 0.768 (95% CI 0.684–0.838) and 0.779 (95% CI 0.696–0.848) in the development cohort, respectively (Fig. 3a). The combined model that integrated two modalities showed an improved AUC of 0.848 (95% CI 0.774–0.906) compared with that of the DWI model (*P* = 0.126) and ADC model (*P* = 0.070) in the development cohort. There were no statistically significant differences in AUCs between the DWI model and ADC model (*P* = 0.890). As shown in Fig. 3b, similar results were obtained in the test cohort. Although not statistically significant, the AUC of the combined model [0.767 (95% CI 0.613–0.882)] was higher than that of the DWI model (*P* = 0.550) and ADC model (*P* = 0.626). The detailed performance of the DL models in the development and test cohorts was summarized in Tables 2 and 3, respectively.

**Table 2:** Performance comparison of the predictive models in the development cohort

Model	AUC (95% CI)	*P*-value	Sensitivity (%)	Specificity (%)	PPV (%)	NPV (%)
ADC	0.779 (0.696–0.848)	*0.025*	83.3	65.6	43.1	92.6
DWI	0.768 (0.684–0.838)	*0.039*	60.0	86.5	58.1	87.4
Combined	0.848 (0.774–0.906)	*0.315*	83.3	72.9	49.0	93.3
Nomogram	0.890 (0.821–0.938)	*Reference*	73.3	93.8	78.6	91.8

*AUC* Area under the curve; *CI* Confidence interval; *PPV* Positive predictive value; *NPV* Negative predictive value; *ADC* Apparent diffusion coefficient; *DWI* Diffusion-weighted imaging

*P* < 0.05 indicates statistically significant

**Table 3:** Performance comparison of the predictive models in the test cohort

Model	AUC (95% CI)	*P*-value	Sensitivity (%)	Specificity (%)	PPV (%)	NPV (%)
ADC	0.719 (0.561–0.845)	*0.347*	54.6	87.5	60.0	84.8
DWI	0.724 (0.567–0.849)	*0.137*	63.6	81.3	53.8	86.7
Combined	0.767 (0.613–0.882)	*0.427*	63.6	81.3	53.8	86.7
Nomogram	0.844 (0.701–0.936)	*Reference*	72.7	90.6	72.7	90.6

*AUC* Area under the curve; *CI* Confidence interval; *PPV* Positive predictive value; *NPV* Negative predictive value; *ADC* Apparent diffusion coefficient; *DWI* Diffusion-weighted imaging

*P* < 0.05 indicates statistically significant

### Development and assessment of the nomogram

**Fig. 4:**
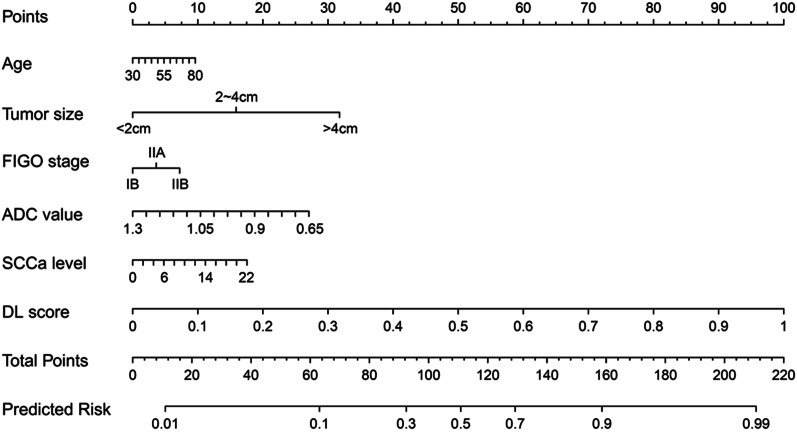
The nomogram combining age, tumor size, FIGO stage, ADC value, SCCa level and the DL score

**Fig. 5:**
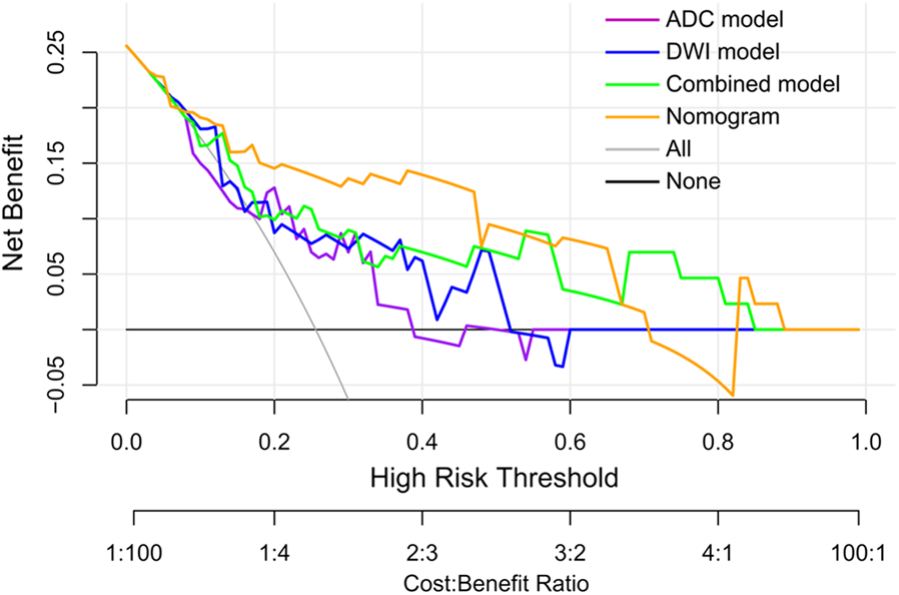
Decision curve analysis for the predictive models and the nomogram. The DL nomogram showed a higher overall net benefit than the other models across the majority of the threshold range

To incorporate the predictive results of the combined model and the clinical characteristics of patients, a nomogram was constructed using the DL score which was the metastasis probability predicted by the combined model, as well as age, tumour size, FIGO stage, ADC value and SCCa level of the patients (Fig. 4). The nomogram showed the best performance with achieving the AUC of 0.890 (95% CI 0.821–0.938) in the development cohort (Fig. 3a), which outperformed the single modality MRI models (*P* = 0.039 vs. DWI model and *P* = 0.025 vs. ADC model) and was also higher than that of the combined model (*P* = 0.315). In the test cohort, the nomogram that integrating clinical variables also showed improved performance than the DL models (Fig. 3b), with AUC increased to 0.844 (95% CI 0.701–0.936). The detailed performance comparisons between the DL models and the nomogram were presented in Tables 2 and 3. The regression coefficients of the predictors used in the nomogram were summarized in Table 4. Furthermore, the DCA demonstrated that the nomogram had a higher overall net benefit than the other models across the majority of the threshold range (Fig. 5).

**Table 4:** Regression coefficients of the variables in the nomogram

Variable	Coefficient	Std. error	Wald	*P*-value
Age	0.009	0.027	0.109	*0.741*
Tumor size	0.731	0.498	2.157	*0.142*
FIGO stage	0.166	0.516	0.104	*0.747*
ADC value	− 1.917	2.328	0.678	*0.410*
SCCa level	0.037	0.035	1.127	*0.288*
DL score	4.603	0.953	23.353	*< 0.001*

*FIGO* International Federation of Gynecology and Obstetrics; *ADC* Apparent diffusion coefficient; *SCCa* Squamous cell carcinoma antigen; *DL* Deep learning

*P* < 0.05 indicates statistically significant

### Interpretation of the combined model

**Fig. 6:**
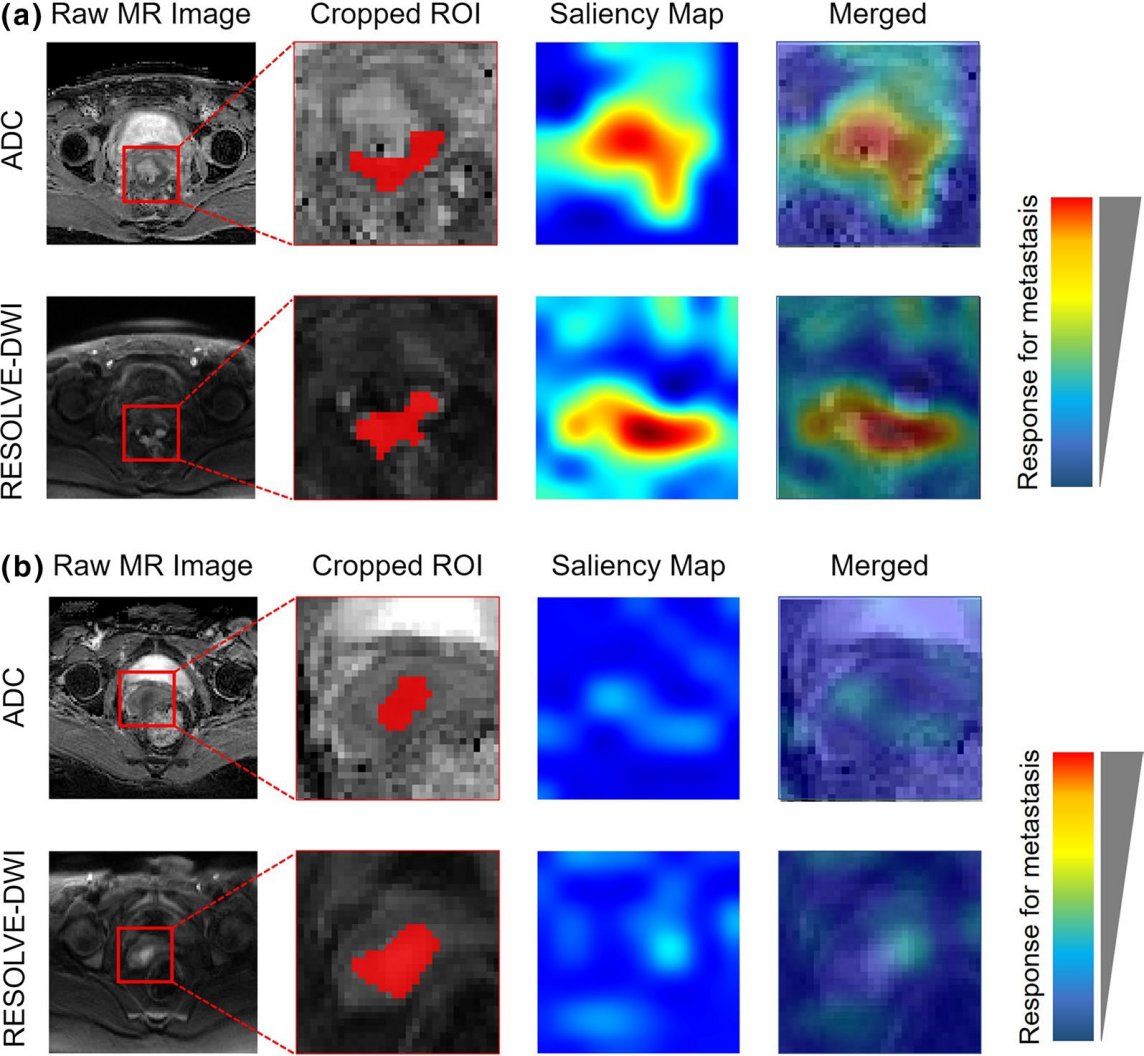
Saliency maps for the combined model. The ADC and RESOLVE-DWI modalities of the MR images and the corresponding saliency map of a metastasis example (**a**) and a non-metastasis example (**b**) were shown, respectively. The red color highlighted the activation region associated with the prediction of metastasis or non-metastasis

To examine the features learned from the MR images by our DL models, we had visualized the most salient regions used by the combined model to discriminate metastasis from non-metastasis. According to the network structure, class-specific activation maps on MRI slices were generated by using Grad-CAM method (Fig. 6). These saliency maps highlighted the regions of visual features in the MR images that responded to the prediction for lymph node metastasis, as the highlighted regions were found in the metastasis patient (Fig. 6a) but not the non-metastasis patient (Fig. 6b). In addition, the saliency map of the metastasis patient (Fig. 6a) also suggested that both intra-tumoral and peri-tumoral regions could contributed to normal-sized LNM prediction in cervical cancer.

## Discussion

In this study, a non-invasive DL nomogram model based on RESOLVE-DWI and clinical information was developed to preoperatively predict normal-sized LNM in cervical cancer. The combined model that integrated RESOLVE-DWI and ADC maps performed better than the two models based on single-modality MR images. The DL nomogram based on the combined model, as well as age, tumour size, FIGO stage, ADC value, and SCCa level, showed the best performance, yielding AUCs of 0.890 and 0.844 in the development and test cohorts, respectively.

For preoperative imaging evaluations of LNM in cervical cancer, positron emission tomography (PET) has the highest specificity, while DWI has the highest sensitivity [23]. Considering that PET has not been widely used in clinics thus far, DWI may be the optional method. Especially for normal-sized LNM with occult micrometastasis, the DWI and ADC maps can provide potential information on the tumour microstructure. However, conventional DWI based on SS-EPI is considered a poor display of anatomical structures and lesions due to its sensitivity to susceptibility to artefacts and geometric distortion, affecting reader confidence in the evaluation of primary tumours or lymph nodes. Nevertheless, the RESOLVE-DWI method used in this study can overcome these inherent shortcomings by segmenting the k-space into several shots along the readout direction to shorten the echo spacing and provide improved image quality in cervical cancer [17]. In our preliminary study, the AUCs of DL models based on RESOLVE-DWI and ADC maps in predicting normal-sized LNM in cervical cancer were 0.724 and 0.712 in the test cohort, respectively. This result is similar to previous studies based on traditional single-modality DWI for predicting enlarged LNM in cervical cancer [24, 25]. Moreover, the combined DL model showed a better performance, achieving an AUC of 0.767 in the test cohort. All of these results suggested that the DL model based on RESOLVE-DWI has the potential to predict normal-sized LNM in cervical cancer.

In addition to image information, clinical information is also important for the preoperative prediction of normal-sized LNM [26]. The clinical information incorporated in previous studies [10, 15, 16, 24, 25] mainly included general information (such as age, menstrual status, FIGO stage, and SCCa level), radiological information (such as MRI-reported LNM and tumour diameter) and histological information (such as differentiation grade, depth of invasion, parametrial or margin involvement, and lymphovascular invasion). These factors may be associated with the risk of LNM in cervical cancer. It should be noted that these postoperative pathological features are not suitable for integration into the preoperative model because not all of these factors are available for every patient [27]. Therefore, we only integrated the explicitly factors preoperatively available through non-invasive methods in our prediction model. In our study, a DL nomogram was constructed by incorporating age, tumour size, FIGO stage, ADC value, SCCa level and DL score. The model achieved a satisfactory result for predicting normal-sized LNM in cervical cancer, with an AUC of 0.890 in the development cohort and 0.844 in the test cohort. This model further improved the diagnostic efficiency over that of the DL models based on single-modality MR images alone, which was consistent with previous studies [10, 15, 16, 24, 25].

Nomogram transforms a complex regression equation into a visual graph and can incorporate different predictive indicators to make the prediction model more readable and convenient for patient evaluation and assist in personalized treatment decision-making. The DL nomogram in our study showed slightly improved performance compared with the best DL model, although the difference was not statistically significant. However, we noted that the specificity of the DL nomogram in predicting LNM obviously increased (to over 90%) after integrating preoperative indicators. This result suggested that the incorporation of MR images and clinical information into the nomogram may be mutually beneficial. In particular, the inclusion of both types of data may help improve the relatively low specificity of the DWI sequence for identifying small metastatic lymph nodes.

Compared with other previous studies [28–32] that have used MR images to detect normal-sized LNM in patients with cervical cancer, our study has four main advantages. Firstly, we outlined and extracted image information based on primary tumours rather than lymph nodes [29–31]to promote to the clinical simplicity and operability. On the one hand, it is difficult to evaluate every lymph node on cross-sectional images because of the limitations of imaging range and lymph node size [12]. On the other hand, LNM is fundamentally driven by the primary tumour, and image-based DL analysis of primary tumours may reveal characteristics that relate to the risk of LNM [33]. Secondly, this is the first study using RESOLVE-DWI as the input of the DL model. Compared with other conventional sequences [31, 32], DWI can improve tumour delineation in cervical cancer and reduce disagreement between observers to avoid the potential pitfalls of discerning tumours from peritumoral oedema [34]. Thirdly, the predictive factors in our model were objective and easy to obtain preoperatively for every patient. Lymph node appearance, shape, border and signal characteristics have been demonstrated to be related to metastasis [28, 29, 31], but these subjective factors may suffer from personal subjectivity and relatively low detection capability [35]. Fourthly, in our study, we extracted and utilized DL features for model building and visualized the most salient regions on MR images that responded to the relevant prediction. It has been reported that the robustness and reproducibility of MRI-derived radiomics features is still a critical problem and may affect their application in clinics [36]. Compared with radiomics [31], DL is more accessible for clinicians because it jointly performs the processes of data representation and prediction without precise tumour segmentation, which simplifies the analysis procedure [25, 34]. In addition, unlike radiomics features, DL features were computed from both the tumour area and its surrounding tissue, which led to the development of a better model [37]. In our study, we also found that the activation region associated with the prediction consisted of both intratumoral and peritumoral tissues of cervical cancer, which has been proven to be an important area for LNM prediction [24, 25].

There are some limitations in our study. Firstly, it was a single center study with a limited number of cases. Although we used image augmentation and transfer learning to improve the model performance and reduce the risk of overfitting, more data from multiple centers for external validation is needed. Secondly, we only preliminarily evaluated the potential of RESOLVE-DWI. The combination of other sequences or other imaging methods may further improve the discriminative performance of the model. Thirdly, our prediction model is based on patients, which has limitations in predicting LNM at node or region level. More prospective studies for personalized and precise clinical tasks will be worthwhile.

## Conclusion

A DL nomogram based on RESOLVE-DWI and related patient information we developed has the potential to predict normal-sized LNM in cervical cancer. This may help clinicians to facilitate preoperative decision-making.

## Data Availability

The datasets used and analysed during the current study are available from the corresponding author on reasonable request.

## Abbreviations

LNM: Lymph node metastasis
DL: Deep learning
RESOLVE-DWI: Readout segmentation of long variable echo-trains diffusion weighted imaging
ADC: Apparent diffusion coefficient
FIGO: International Federation of Gynecology and Obstetrics
SCCa: Squamous cell carcinoma antigen
CNN: Convolutional neural network
SS-EPI: Single-shot echo-planar imaging
Grad-CAM: Gradient-weighted class activation mapping
DCA: Decision curve analysis
